# CENP-A: the key player behind centromere identity, propagation, and kinetochore assembly

**DOI:** 10.1007/s00412-012-0386-5

**Published:** 2012-10-26

**Authors:** Valérie De Rop, Abbas Padeganeh, Paul S. Maddox

**Affiliations:** Institute for Research in Immunology and Cancer (IRIC), Department of Pathology and Cell Biology, Université de Montréal, C.P. 6128, succursale Centre-ville, Montréal, Québec H3C 3J7 Canada

## Abstract

Chromosome segregation is the one of the great problems in biology with complexities spanning from biophysics and polymer dynamics to epigenetics. Here, we summarize the current knowledge and highlight gaps in understanding of the mechanisms controlling epigenetic regulation of chromosome segregation.

## Introduction

Centromeres were first described by Walther Flemming as the primary constriction on condensed chromosomes, where cellular fibers, now known to be microtubules, attached during mitosis (Flemming 1882). Electron microscopy images later led to the realization that centromeres are chromosomal loci where the megadalton protein complex named the kinetochore assembles (Robbins and Gonatas 1964; Brinkley and Stubblefield 1966). The definition of centromeres, while still following these early rules, has become more complex with the discovery of molecular players involved in centromere identity. Counter intuitively, extensive studies on the expression and localization of different centromere components throughout the cell cycle have not led to a consensus mechanism that defines centromere specification. In this review, we focus on the latest discoveries in the field and specifically on epigenetic markers for centromere identity.

## CENP-A as an epigenetic marker for centromere identity

Centromeres are epigenetically defined by a variant of histone H3, centromere protein-A (CENP-A). CENP-A was serendipitously discovered in 1985 by William Earnshaw in the course of immunoblotting and immunostaining experiments. Blotting serum isolated from CREST syndrome patients identified three recurrent bands common among many patients. Immunostaining using the same sera in tissue culture cells showed an enrichment at centromeres (Earnshaw and Rothfield 1985; Earnshaw et al. 1986; Valdivia and Brinkley 1985). These proteins were accordingly named centromere proteins A, B, and C. Later, biochemical approaches demonstrated that CENP-A copurified with histones and is a bona fide component of nucleosome particles (Palmer et al. 1987; Palmer et al. 1991). CENP-A contains a histone fold domain and is able to replace histone H3 in centromeric nucleosomes (Sullivan et al. 1994; Yoda et al. 2000). Sequence analysis of CENP-A and histone H3 reveals a 60 % similarity within the histone fold domains with major differences concentrated in the N-terminus (Sullivan et al. 1994). Through the following years, CENP-A homologues were identified in all eukaryotic model systems investigated: for example, HCP-3 in *Caenorhabditis elegans*, CID in *Drosophila melanogaster*, Cse4 in *Saccharomyces cerevisiae*, Cnp1 in *Schizosaccharomyces pombe*, and CenH3 in *Arabidopsis thaliana* (Buchwitz et al. 1999; Blower and Karpen 2001; Stoler et al. 1995; Takahashi et al. 2000; Talbert et al. 2002). Interestingly, CENP-A is poorly conserved compared to other histone proteins which are almost invariant through evolution at the amino acid level. Divergence, while extreme in the N-terminal tail of CENP-A, is prevalent even within the C-terminal histone fold of closely related species. Even if CENP-A is poorly conserved at the sequence level, its structure or active chemical tags (i.e., acetylation and methylation) may be specific features that are keys to epigenetic mechanisms controlling chromosome segregation.

Understanding differences between CENP-A and histone H3 has long been thought to hold the answer to epigenetic regulation of centromeres. In an attempt to understand the differences in molecular dynamics between the two histone proteins, the Cleveland lab identified a specific domain of CENP-A which they called the CENP-A targeting domain (CATD) using a hydrogen/deuterium exchange technique coupled to mass spectrometry (H/DX-MS). With this technique, they showed less deuterium exchange in the CATD domain of CENP-A and thus concluded that the CATD is less flexible and more compacted (Black et al. 2004; Black et al. 2007a). Remarkably, the CATD domain was shown to be sufficient for CENP-A localization to centromeres as demonstrated by swapping the CATD domain from CENP-A in histone H3 chimeric proteins (Black et al. 2007b). This exciting result was later shown to be due to recognition by a centromere-specific chaperone protein, Holiday junction-recognizing protein (HJURP, see below) (Bassett et al. 2012). Consistent with protein structure playing a critical role, HJURP binding to CENP-A-H4 induces more compaction and less flexibility compared to a control condition. Thus, it is clear that structural distinction in CENP-A is essential for centromere identity and function.

Subsequent structural studies have yielded a more precise understanding of the atomic differences between H3 and CENP-A nucleosomes. For instance, the CENP-A-H4 tetramer crystal obtained by the Black lab showed unique biophysical properties of CENP-A nucleosomes (Sekulic et al. 2010). Overlay of H3-H4 and CENP-A-H4 tetramers revealed that the centromere-specific tetramer is rotated between dimer pairs compared to H3-H4 tetramer. This rotation is caused by two specific residues, His104 and Leu122, located at the CENP-A/CENP-A interface. Also, these residues were shown to be essential for CENP-A localization to the centromeres. Moreover, this rotation makes the CENP-A-H4 tetramer more compacted and less flexible as demonstrated by H/DX-MS. Overall, these features make the CENP-A-H4 tetramer narrower and shorter in 2D and wider in 3D compared to H3-H4 tetramers. In addition, the same group demonstrated a bulged structure in the L1 loop conferred by the Arg80 and Gly81 residues. Concomitantly, Kurumizaka’s group also indentified these residues and showed their importance in the stability of CENP-A localization (Tachiwana et al. 2012). This group solved the CENP-A nucleosome crystal structure and discovered that the CENP-A αN is shorter than that of histone H3, although they did not demonstrate the importance of this structural feature. All together, these results clearly demonstrate significant structural differences between CENP-A and H3 derived octamers.

The octamer structure is not the only source of distinction between CENP-A and H3. Recent studies of DNA wrapping topology by H/DX-MS showed that residues causing structural deformation are found in the αN part of CENP-A sequence adjacent to the DNA entry–exit site of the nucleosome (Panchenko et al. 2011). In canonical chromatin, this site is known to be recognized by diverse functional proteins, e.g., for transcriptional control by stabilization of nucleosomes, inhibition of nucleosome sliding, and compaction of chromatin in mitosis (Zlatanova et al. 2008). Even more radical differences in DNA wrapping have been reported. Henikoff’s group provided evidence suggesting that centromeric DNA, instead of having a left-handed wrapping around nucleosomes with negative supercoiling, as for canonical H3 nucleosomes, is in fact wrapped in a right-handed manner causing positive supercoils or less negative supercoils (Furuyama and Henikoff 2009). This was shown biochemically using an extrachromosomal plasmid and determining the state of DNA by inducing structural deformation by chloroquine-infused gel electrophoresis. In summary, on one hand, we now have a good understanding of the structural differences between CENP-A and H3 nucleosomes, while on the other hand, new questions are arising regarding the precise topology of centromeric chromatin. Some progress has been made in the last year to understand if the observed structures are species and/or cell cycle specific (Bui et al. 2012; Shivaraju et al. 2012). Nonetheless, it is still controversial whether those structures are critical for CENP-A loading, incorporation, and stabilization at centromeres.

## Does DNA sequence have any role in centromere propagation and identity?

**Fig. 1:**
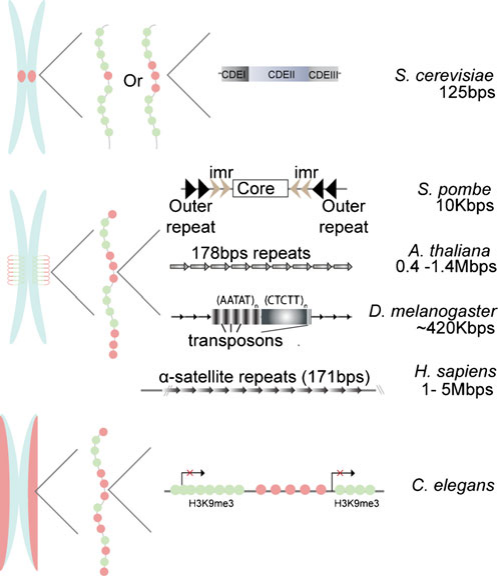
Centromeric DNA sequences are not conserved through species. With the exception of *S*. *cerevisiae*, most evidence shows that DNA sequence has no role in centromere identity. Species comparison reveals vast differences in nucleotide composition as well as the centromere length. *S*. *cerevisiae* has a 125-bp centromere which is divided in three centromere DNA elements (CDE), with CDEII being the sequence important for Cse4 incorporation. It is still somewhat controversial whether this centromere is composed of a single or two to three CENP-A nucleosomes.(Lawrimore et al. 2011; Furuyama and Biggins 2007) *S*. *pombe* 10 kbp centromere consists of inner and outer repeats located outside the core region and in a head-to-head orientation. *D*. *melanogaster* has relatively large centromeres made up of DNA repeats and transposon elements for a genomic size of ∼420 kbp. *A*. *thaliana* and *Homo sapiens* centromeres are made of head-to-tail 171–178 bp repeats that can go up to 1.4 Mbp for the plant and 5 Mbp for human. General centromere structures display two general types of centromere: monocentric for *S*. *cerevisiae* to *H*. *sapiens*, and holocentric (whole length of chromosome) for *C*. *elegans* (generally nematodes and several other species) (Maddox et al. 2004; Melters et al. 2012) Elongated centromeric chromatin may have different arrangements of CENP-A and H3 nucleosomes arrays, which are repetitive and exclusive from one another (Blower et al. 2002). When compacted, the CENP-A arrays form a hypothetical centromeric plate required for kinetochore formation in mitosis. *Green circles* H3 nucleosome, *red circles* CENP-A nucleosome

**Fig. 2:**
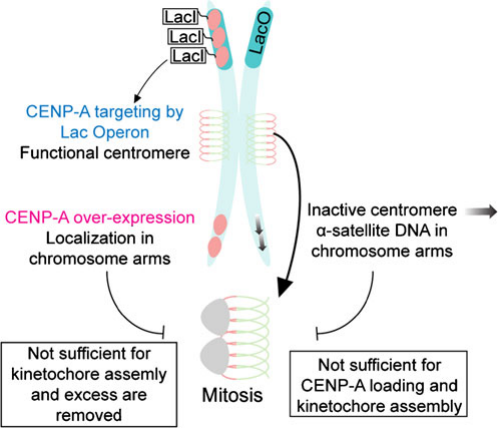
Compacted CENP-A chromatin promotes kinetochore assembly and centromere propagation. An inactive centromere, which is a chromosomal locus having alpha-satellite sequence without CENP-A protein, demonstrated that centromeric sequence is not sufficient for kinetochore assembly. Kinetochore proteins are recruited to this chromosomal locus only when CENP-A is present (active centromere) (Warburton et al. 1997). Conversely, when CENP-A is overexpressed in cells, it incorporates on chromosome arms. However, this mislocalized CENP-A does not recruit kinetochore proteins in mitosis. Ultimately, by targeting CENP-A on chromosome arms using the lac operon technique, this creates a high-density array of CENP-A protein and forms a neocentromere: it recruits kinetochore components and is able to propagate for a limited time. Those observations suggest that CENP-A density is important for centromere identity and its essential role of kinetochore assembly

For decades, centromere identity has been thought to rely on an epigenetic mechanism due to a myriad of experimental evidences, with the exception of *S*. *cerevisiae*. Centromere sequences are highly divergent throughout species (Fig. 1). In *S*. *cerevisiae*, centromeres are 125 bp and composed of three centromere DNA elements (CDEI-III). A single mutation in the CDEII element abrogates Cse4 incorporation and leads to a loss of centromere function and cell death (Cottarel et al. 1989; Spencer and Hieter 1992). In *S*. *pombe*, centromeres consist of 40 to 100 kbp of repeated and inverted sequences (Clarke et al. 1986; Fishel et al. 1988). *D*. *melanogaster* centromeres are repeats of transposon and satellite sequences (AATAT and CTCTT) that can measure up to 420 kbp (Murphy and Karpen 1995; Sun et al. 1997). In humans, centromeres are composed of A/T rich α-satellite repeat sequences of 171 bp arranged in a head to tail orientation, ranging in total size from 1 up to 5 Mbp (Tyler-Smith et al. 1993; Manuelidis 1978; Mitchell et al. 1985; Willard 1985). Interestingly, human centromeres vary in genetic length on different chromosomes. The discovery of neocentromeres in humans is the key experimental evidence (provided by the stochastic nature of biology) for epigenetic regulation of centromeres. Found in exceedingly low frequency, neocentromeres form on a unique chromosomal locus distinct, and typically displaced by mega bases, from the “normal” centromere genomic position. This genomic displacement is not due to translocation or rearrangements and represents a novel epigenetic event. Neocentromeres contain CENP-A nucleosomes and are able to direct assembly of functional kinetochores supporting normal development, all in the absence of α-satellite DNA (Warburton et al. 1997). In addition, considering that α-satellite sequences can be found in non-centromeric loci (called inactive centromere), it is assumed that DNA sequence is not sufficient to incorporate CENP-A and build a functional kinetochore (Van Hooser et al. 2001; Earnshaw et al. 1989; Haaf et al. 1992; Warburton et al. 1997). To demonstrate this point, experiments performed by Earnshaw’s group on dicentric chromosomes showed that only the active centromere, and not an inactive one, can build a functional kinetochore (by recruiting CENP-C and nucleating kinetochore assembly) even if the two centromeres are composed of α-satellite DNA (Fig. 2). In sum, all observations lead to the conclusion that centromeric DNA is neither necessary nor sufficient for centromere identity (with the noteworthy exception of *S*. *cerevisiae*).

There is a clear distinction to make between centromere propagation and de novo formation. Recently, evidence has emerged to indicate a role for centromeric DNA sequences in de novo formation of centromeres. Human alphoid DNA repeats found at centromeres can be classified into two types: α21-I and α21-II, with α21-I containing a 17-bp sequence called the CENP-B box (Ikeno et al. 1994). The CENP-B box is recognized and bound specifically by CENP-B, the only centromeric protein recognizing the centromeric DNA (Masumoto et al. 1989; Muro et al. 1992). Masumoto’s group demonstrated that the CENP-B box together with alphoid DNA sequence is sufficient for de novo centromere formation in human tissue culture cells. These artificial centromeres contain CENP-A and are competent to recruit kinetochore components such as CENP-C and CENP-E (Ohzeki et al. 2002). *C. elegans* sperm DNA is known to be deficient of CENP-A protein before fertilization and gains it after entering the oocyte. Despite the lack of CENP-B protein in worms, the sperm DNA is able to form a new centromere via an unknown molecular mechanism (Gassmann et al. 2012). Another study showed that the Aurora B mitotic kinase, an inner centromere protein known to be required for correcting abnormal kinetochore–microtubule attachments, decreases at neocentromeres, concluding that this chromosomal environment, for an unknown reason, is less favorable for inner centromere maturation (Bassett et al. 2010). Hypothetically, decreased inner centromere assembly could be due to the DNA sequence itself, or it could also be that alphoid DNA sequences which are A/T rich could promote a more compacted chromatin structure that is favorable for inner centromere assembly (Ganter et al. 1999; Dlakic and Harrington 1996). In summary, the precise role of centromeric DNA is still unclear; however, the fact that there is centromeric DNA signatures indicates that these genomic regions are important for centromere function.

## A role for CENP-A nucleosome composition in centromere propagation

CENP-A nucleosome composition has become a fascinating debate. Some pieces of evidence demonstrate that CENP-A is an octamer while another scenario proposes it is half an octamer, a hemisome, or a tetrasome (Camahort et al. 2009; Palmer et al. 1987; Sekulic et al. 2010; Dalal et al. 2007; Williams et al. 2009; Mizuguchi et al. 2007) and reviewed in (Black and Cleveland 2011; Henikoff and Furuyama 2012). Here, we specifically describe how CENP-A nucleosome composition could be dynamic through the cell cycle and how this feature can manifest in conserved centromere identity over repetitive cell divisions.

In 2007, two key papers showed that CENP-A incorporation occurs after mitotic exit and takes place from late anaphase to early G1 (Schuh et al. 2007; Jansen et al. 2007). Using live cell imaging coupled to quantitative super-resolution analysis, we showed that CENP-A loading in human cells takes the entire length of G1 (about 8 h) (Lagana et al. 2009). Interestingly, in plants, CENP-A seems to be loaded in G2 as shown in two examples: *A*. *thaliana* and *Hordeum vulgare* (Lermontova et al. 2007; Lermontova et al. 2011). Regardless of the precise timing of CENP-A incorporation, it universally occurs outside of S-phase leading to a centromere identity problem in the next cell cycle. After the passage of the replication fork, canonical nucleosomes are randomly distributed onto replicated daughter strands, leaving gaps that are filled with newly synthesized histone proteins (H3, H4, H2A, and H2B) (Smith and Stillman 1991). CENP-A nucleosomes should also be randomly distributed, but gaps will be left after the replication fork passage since newly synthesized CENP-A is not loaded in S-phase, thus diluting the epigenetic mark. Karpen’s group tackled this problem and discovered that histone H3.3, another non-replication coupled histone H3 variant, fills in the gaps until the next G1 phase where it is specifically replaced by CENP-A (Dunleavy et al. 2011). Thus, centromeric chromatin is thought to be stabilized and reinforced by a combination of H3 variants in S-phase. An alternate model proposed by Henikoff’s group relies on evidence of CENP-A hemisomes (tetramers of CENP-A/H4/H2A/H2B) at centromeres, supported by atomic force microscopy (AFM) analysis showing centromeric nucleosomes having half the height of a canonical octameric nucleosome (Dalal et al. 2007). The model suggests that CENP-A nucleosomes are split in half on each daughter strands in S-phase, keeping the centromeric epigenetic mark and the size of the centromere locus for each cell division. This is an attractive model for centromere identity preservation, which does not include an intermediate centromeric composition. However, a hemisome model does not take into account the packing of DNA as hemisomes will wrap approximately half the length of DNA compared to octamers.

Even if the hemisome model is controversial, it raises the possibility that CENP-A nucleosome composition is dynamic through the cell cycle. Recently, two papers concluded that CENP-A chromatin dynamically switches between octamer and tetramer (CENP-A-H4-H2A-H2B) compositions in different phases of the cell cycle (Bui et al. 2012; Shivaraju et al. 2012). Dalal’s group measured nucleosome height and volume using AFM and they demonstrated that CENP-A nucleosomes are tetramers in G1, convert into octamers in early S, and revert back into tetramers at the end of S-phase. Whereas the Gerton lab, using fluorescence correlation spectroscopy (FCS) coupled to calibrated imaging, observed that yeast centromeres have one copy of Cse4 during the majority of the cell cycle and two copies at anaphase B. There are caveats with these results however. AFM data are based on affinity purification of centromeric nucleosomes whose precise biochemical makeup is not clear. Thus, differences in height could be attributed to the presence of additional proteins or other artifacts generated in the purification scheme. FCS is a powerful technique that uses peak fluorescence intensity from a diffraction limited spot (less than 300 nm in this case) to determine protein concentration and diffusion rates. However, yeast centromeres are often dispersed (especially in metaphase) and do not all occupy a diffraction limited spot, which would result in an underestimation of Cse4 number. During anaphase, yeast centromeres are highly compacted fitting nicely into a diffraction limited spot. This topographical difference would nicely explain the FCS results. Regardless, centromere identity and composition are necessarily dynamic because of genome replication. It will be of great interest to determine if these observations are born out by the test of time.

## CENP-A loading onto centromeric chromatin is a three-step mechanism

It has been clearly demonstrated in virtually every model system that nucleosome components (H3, H4, H2A, and H2B) are expressed in S-phase (Prescott 1966; Borun et al. 1975). Nucleosome assembly occurs in an ordered manner with the help of chaperone proteins, such as CAF-1 and Asf1, and the resultant octamers are formed after the replication fork (Smith and Stillman 1991, 1989; Hayashi et al. 2004). However, CENP-A, like other histone variants, has a distinct expression pattern. Human CENP-A mRNA peaks in G2 prior to mitosis, and its incorporation is restricted to G1 phase as discussed above (Shelby et al. 2000; Jansen et al. 2007; Schuh et al. 2007; Hemmerich et al. 2008). CENP-A incorporation in this window of the cell cycle is very interesting and raises many questions. One model wherein mitotic forces transmitted through kinetochore microtubule attachments somehow modify centromere structure was disproven by bypassing the spindle assembly checkpoint (O'Connell et al. 2008). Centromeres that never experienced forces (due to absence of microtubules) incorporated new CENP-A with normal G1 timing (Jansen et al. 2007; Schuh et al. 2007). Thus, the cellular mechanism propagating centromere identity in G1 is still largely obscure on a cell biological level.

**Fig. 3:**
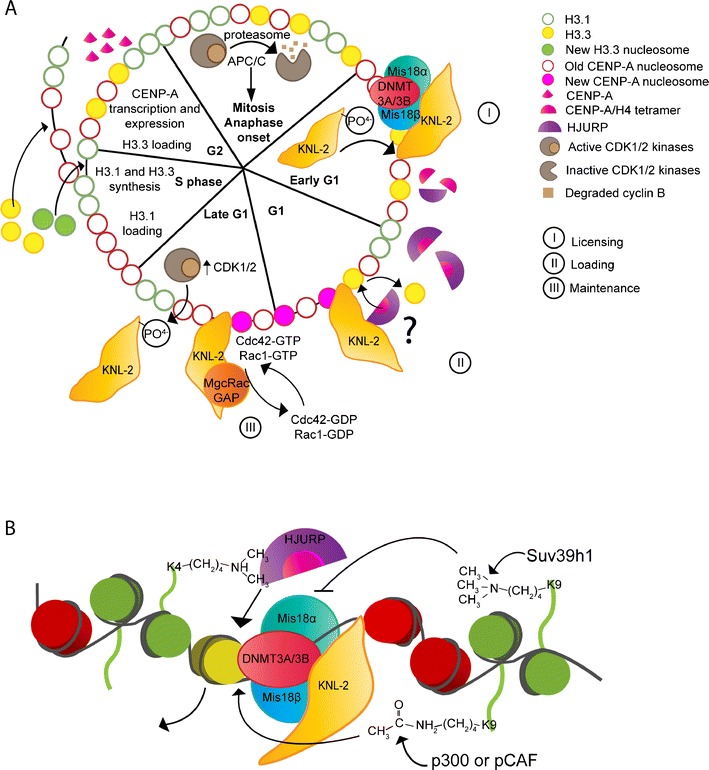
CENP-A incorporation is cell cycle regulated and depends on epigenetic marks. **a** CENP-A incorporation is a replication-independent mechanism; CENP-A-H4 tetramers are loaded in G1 instead of S-phase as for H3-H4 tetramers. The incorporation is a three-step mechanism: I—Licensing, II—Loading, and III—Maintenance. First, the licensing complex (KNL-2, Mis18α and β) recognizes and binds the centromeric chromatin. This will license the centromere for loading of newly synthesized CENP-A. Next, the licensing complex recruits, by an unknown mechanism, the CENP-A chaperone HJURP which directly binds and stabilizes CENP-A/H4 complexes. Finally, when newly synthesized CENP-A is incorporated, a mark is removed or CENP-A conformation is changed in order to change the newly synthesized identity to that of an old one. Cdc42 or Rac1 might be part of this cellular process through the action of the GAP MgcRacGAP and the GEF ECT-2. CENP-A loading to centromeres is regulated through the cell cycle by the CDK1/2 kinases, which phosphorylate KNL-2 in mitosis and block its localization to centromeres. At anaphase onset, CDK activity diminishes, KNL-2 is dephosphorylated and is able to localize to centromeres. **b** CENP-A incorporation to centromeres also depends on a post-translational modification of histone H3. H3K4 dimethylation is important to eventually HJURP, which localizes to centromeres and loads newly synthesized CENP-A. H3K9 trimethylation by the methyltransferase Suv39h1 inhibits CENP-A loading to centromeres, whereas H3K9 acetylation by the histone acetyl transferase (HAT) p300 or pCAF triggers CENP-A loading at centromeres. We hypothesize that KNL-2 to binds the linker DNA in the centromeric chromatin and, together with its partners Mis18α and Mis18 β, this protein complex acts as a licensing mark of centromeres for CENP-A loading. Also, a DNA methyltransferase DNMT3A and DNMT3B interacts with Mis18α, and depletion of the protein leads to a decrease of other epigenetic marks such as H3K9me2, H3K9m3, and H3K4me2 at centromeric loci

On the molecular level, several studies have addressed the mechanism of CENP-A loading onto centromeric chromatin. Currently, a three-step mechanism loosely describes the process: (1) recognition and licensing of centromeres, (2) loading of newly synthesized CENP-A with the help of chaperone proteins, and (3) maintenance of newly incorporated CENP-A (Fig. 3a).

1. Recognition and licensing of centromeres
  Recognition of centromeric chromatin for CENP-A loading only at centromeres is a complex question. In 2007, a licensing complex, also called the Mis18 complex, consisting of KNL-2 (M18BP1, hereafter referred to as KNL-2) and its partners Mis18α/β was shown to be required for CENP-A localization to centromeres in diverse model systems (Fujita et al. 2007; Maddox et al. 2007). Our understanding of the mechanism of the licensing complex is limited; however, it is clear that its recruitment to centromeres following anaphase is the most upstream event known for CENP-A deposition. It has been proposed that CENP-C recruits the licensing complex (Moree et al. 2011; Dambacher et al. 2012). This model is based on the observation that KNL-2 had reduced localization at centromeres when CENP-C was depleted in *Xenopus* egg extracts (Moree et al. 2011). However, KNL-2 localization is not fully lost and CENP-C is clearly downstream of KNL-2 in other model systems (Maddox et al. 2007; Fujita et al. 2007). Thus, the question of how the licensing factors recognize and bind centromeric chromatin with high specificity and subsequently recruit downstream components required for CENP-A loading needs more investigation.
2. Loading of newly synthesized CENP-A with the help of chaperone proteins
  There have been two factors identified that stabilize the CENP-A/H4 complex, RbAp46/48 (Mis16, a member of the CAF-1 complex) and HJURP (or SCM3 in yeast). These essential proteins were shown to be required to prevent the degradation of soluble CENP-A molecules and thus considered chaperones (Foltz et al. 2009; Dunleavy et al. 2009; Hayashi et al. 2004). As RbAp46/48 seems to be a nonspecific histone chaperone, research has focused on HJURP showing it to have nucleosome assembly activity specifically for newly synthesized CENP-A (Dunleavy et al. 2009; Foltz et al. 2009; Barnhart et al. 2011). The CENP-A binding domain in the N-terminus of HJURP recognizes the CATD, localized in the histone fold domain within the L1 and α2, of CENP-A (Shuaib et al. 2010). Further details of the nature of this interaction were uncovered by co-structural studies of HJURP and CENP-A/H4, identifying specific residues mediating direct binding (Hu et al. 2011b). Although it is well accepted that HJURP recognizes CENP-A through CATD binding, in 2012, the Black lab discovered another binding interface located in the N-terminus of CENP-A (Bassett et al. 2012). This distinct interface is not required for the specificity of the binding but rather for stabilization, a mechanism that results in changing the structure of the histone fold domains of both CENP-A and H4. H3 nucleosome assembly order is well described in the literature; a histone H3/H4 tetramer first sits on the DNA and then two H2A/H2B dimers complete the assembly (Smith and Stillman 1991). However, some biochemical and structural studies show that HJURP binds a dimer of CENP-A/H4 (Hu et al. 2011a). Thus, it is unclear which CENP-A/H4 complex (dimer or tetramer) gets incorporated in the centromeric chromatin. Regardless of the stoichiometry, HJURP localization to centromeres in G1 is dependent on the licensing complex; however, direct binding of the licensing complex components and HJURP has not been observed (Barnhart et al. 2011; Lagana et al. 2009; Fujita et al. 2007).
3. Maintenance of newly incorporated CENP-A
  Immunoprecipitation of KNL-2 allowed our group to identify a new protein involved in centromere identity, MgcRacGAP (Lagana et al. 2009). This GTPase activating protein was previously described as part of the centralspindlin complex with a role in cytokinesis via regulation of Rho-type small G-proteins (Mishima et al. 2002; Canman et al. 2008). Nevertheless, two independent labs had revealed a possible role for MgcRacGAP in centromere function (Izuta et al. 2006; Perpelescu et al. 2009). In our study, we showed that MgcRacGAP localizes to centromeres in late G1 for a brief (1 to 2 h) window following CENP-A loading. Depletion of MgcRacGAP resulted in the loss of newly incorporated CENP-A nucleosomes. Interestingly, a GAP dead mutant expressed in HeLa cells showed persistent localization to centromeres, and depletion of ECT-2 (the partner GTP exchange factor protein) recapitulated MgcRacGAP depletion. Thus a small GTPase cycle is likely required for maintaining CENP-A at centromeres. By depleting known small GTPases, we identified Cdc42 and Rac1 as possible targets of MgcRacGAP and ECT-2, and localization studies led us to favor Cdc42 as the relevant small GTPase. Since the localization of MgcRacGAP to centromeres is very late in G1 and depletion resulted in loss of centromere stability, we hypothesized that MgcRacGAP maintains the newly incorporated CENP-A and prevents overincorporation of CENP-A. This maintenance mechanism undoubtedly requires downstream effectors that are as yet unidentified.

## Post-translational modifications of newly synthesized CENP-A

The importance of histone post-translational modifications has become clear in genome regulation (Fig. 3b). Some modifications affect gene expression through activation or repression of transcription by changing chromatin compaction and state. In centromeres, histone H3 lysine 4 dimethylation (H3K4me2), a marker of transcriptional activation, was shown to be interspersed with CENP-A nucleosomes on elongated *Drosophila* and human chromatin (Sullivan and Karpen 2004). This post-translational modification was shown to be important for centromere regulation, as increased activity of the demethylase LSD1 at human artificial chromosome (HAC) centromere decreases HJURP localization (Bergmann et al. 2011). Furthermore, loss of H3K4me2 prevents loading of newly synthesized CENP-A to this alphoid DNA, revealing an important role of this post-translational modification for CENP-A localization to centromere. In the same study, a loss of H3K4me2 decreased the transcription of alphoid DNA; however, this was not clearly demonstrated to have a direct role with HJURP localization and CENP-A loading. As post-translational modifications such as H3K4me2 affect chromatin compaction state, the physical topology of centromeric chromatin is likely to be critical for CENP-A localization. Additionally, Masumoto’s group demonstrated that histone H3 lysine 9 trimethylation (H3K9me3) prevents de novo CENP-A assembly on HAC alphoid DNA by tethering Suv39h1 (a methyltransferase) at this specific locus in mammalian cells (Ohzeki et al. 2012). Also, after depleting Suv39h1, an increase in CENP-A at HAC alphoid DNA was observed. On the other hand, tethering of histone acetyltransferases p300 or pCAF increased the acetylation state of H3K9 and also increased CENP-A level at alphoid DNA. This modification is important only for de novo CENP-A assembly, as removal of acetyltransferases from cells did not affect preexisting centromeres over several cell divisions.

Interestingly, the balance between methylation and acetylation of H3K9 is linked generally to transcriptional activity indicating a possible link between with CENP-A localization to centromeres. In support of this hypothesis, the Desai lab demonstrated by genomic studies in *C*. *elegans* that CENP-A is incorporated in regions of low germline transcriptional activity (Fig. 1) (Gassmann et al. 2012). One possible mechanism linking post-translational modification, transcription, and CENP-A localization to centromeres could be explained by the observations that RNA polymerase II together with its associated transcription factors are localized to centromeres and these active proteins increase α-satellite transcripts (Chan et al. 2011). Interestingly, inhibition of RNA polymerase II decreased CENP-C localization to centromeres; however, it is unclear if this is cause or effect.

DNA, as well as histone proteins, can be modified by methylation on cytosines of CpG islands. This state of the chromatin is usually linked to transcription repression due to chromatin compaction (Gros et al. 2012). Interestingly, a DNA methyltransferase enzyme called DNMT3A/3B has been shown to co-localize with Mis18α in mouse embryonic fibroblast cells (Kim et al. 2012). Depletion of Mis18α protein leads to a decrease in centromere DNA methylation as well as a decrease of DNMT3A localization to centromeres. Moreover, this depletion leads to a decrease of some post-translational marks on histone H3 such as H3K9me2, H3K9m3, and H3K4me2 at centromeric locus of chromosomes. However, this study does not demonstrate a direct link between those modifications, transcription of α-satellite DNA, and CENP-A localization to centromeres. All together, there is clear evidence that post-translational modifications of centromeric chromatin affect CENP-A loading. This field of research is blooming and we expect great advances in the near future providing a better understanding of those modifications in centromere identity.

## CENP-A loading to centromere is regulated by cell cycle kinase

The timing of CENP-A loading invokes clear hypotheses of a cell cycle-dependent mechanism. Recently, the Jansen lab nicely demonstrated that cyclin-dependent kinases (CDKs) temporally regulate CENP-A loading to centromeres (Silva et al. 2012). Synchronized human cells treated with roscovitine, a CDK1 and 2 inhibitor, showed apparently normal, however mis-timed, CENP-A assembly in G2. More precisely, they demonstrated a role for CDK1 as the kinase regulating CENP-A loading. Using DT40 avian cell line, which is genetically null for CDK2, and inhibiting CDK1 function chemically, the authors observed an inappropriate CENP-A loading in G2, compared to control DT40 cells. Also, this regulation seems to be at the level of KNL-2, as expression of a mutant form with 24 potential phosphorylation sites changed to alanine also resulted in precocious localization of KNL-2 and downstream CENP-A loading in otherwise untreated G2 cells. This regulation through CDK activity is only true for KNL-2 and did not have any influences on the other licensing complex components. Thus, CENP-A loading is inversely timed relative to mitotic CDK activity and KNL-2 seems to be the most upstream component of the centromere epigenetic regulation pathway (Fig. 3a). It is not clear if these rules will hold true for plants where CENP-A loading is in G2; however, some cell cycle timing mechanism must exist in these models also.

## Is the ultimate mark for centromere identity CENP-A?

**Fig. 4:**
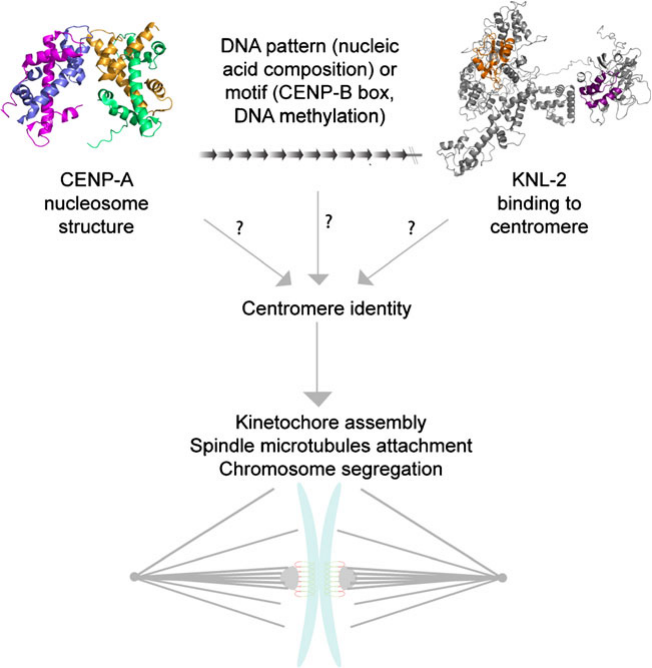
What is the first epigenetic mark of centromeres? Information provided by the literature brings the question of what is the first epigenetic mark of centromeres. Some evidence has shown that CENP-A nucleosome structure might be the feature for centromere identity. However, CENP-A incorporation and localization to centromeres are not passive cellular processes; therefore, CENP-A might not be the first epigenetic mark. Centromere identity could be through genetic features of DNA sequence (nucleic acid composition) or motif (CENP-B box, DNA methylation), but strong experimental evidence disputes this model. One marker that could link the two other hypotheses is KNL-2. This protein was shown to be essential for CENP-A localization to centromeres and genetically, it is the most upstream protein for this important cellular process. Thus, KNL-2 could be the first epigenetic mark of centromeres. For KNL-2 predicted structure: *Orange* is the SANTA domain and in *purple* is the Myb domain. (Published CENP-A nucleosome structure, Protein Data Bank reference # 3AN2 (Tachiwana et al. 2012); KNL-2 predicted structure (Zhang 2008))

The epigenetic mechanism for centromere identity has been well defined over the years. However, only recently was it shown that CENP-A is sufficient to drive centromeric identity over multiple cell divisions (Fig. 4). This was accomplished by generating a high-density region of CENP-A chromatin; it is needed because previous overexpression experiments resulted in CENP-A incorporation in the chromosome arms, however no kinetochore assembly. Briefly, many repeats of the lac operon were integrated in series at a non-centromeric (region on a chromosome arm) locus in flies (Mendiburo et al. 2011). Overexpression of LacI fused to CID (CENP-A) resulted in CID incorporation ectopically, and this ectopic centromere recruited kinetochore components. Remarkably, these ectopic centromeres can, with extremely low fidelity, functionally replace the endogenous centromeres for a short period of time. Similar studies fusing HJURP to LacI in human cells also generated localized high-density arrays of CENP-A with similar results. Interestingly, the lac operon system bypassed the need of the licensing complex for HJURP, since this technique forces direct interactions of the protein with a specific DNA sequence (Barnhart et al. 2011) (Fig. 2). This is a strong argument in favor of the licensing complex being upstream of HJURP for CENP-A loading to centromeres given that HJURP, in the absence of KNL-2 and Mis18, does not localize to normal centromeres. Therefore, high-density CENP-A chromatin is not only necessary but is sufficient for centromere identity and function.

In normal cells, the licensing complex is the first known step in recognizing centromeric. Interestingly, in *C*. *elegans*, it has been observed for quite some time that exogenous DNA injected into oocytes forms stably transmitted arrays that are properly segregated over many generations. Accordingly, the Desai lab reasoned that if this DNA is segregated, then it should form a functional kinetochore (Yuen et al. 2011). To test this hypothesis, they showed that arrays segregate passively until early embryogenesis where at the five to eight cell stages, lacO extrachromosomal arrays recruit centromeric proteins such as CENP-A, kinetochore proteins BUB-1 and NDC-80, and the licensing factor KNL-2, actively segregating the array. This interesting approach demonstrated that random DNA sequences are competent for de novo centromere formation; however, the timing and the sequence of events are not defined. It should be noted that this could be specific to *C*. *elegans* given the fact that alphoid DNA has been demonstrated to be critical for de novo centromere formation in human cells (Ohzeki et al. 2002).

Emerging evidence is expanding our knowledge on the epigenetic mechanism of centromere identity but still raises the question: what is the first mark for centromere identity? We propose two broad possibilities: (1) CENP-A structure and the surrounding post-translational modifications confers to centromere a specific docking site for the licensing complex to bind, loading newly synthesized CENP-A via the chaperone HJURP, (2) centromeric DNA, composed of motifs like the CENP-B box is directly recognized by the licensing complex, or centromeric DNA composition in nucleic acids confers a centromere-specific chromatin state. In either case, we propose that licensing protein KNL-2 through its Myb domain binds the centromeric DNA and recruits the loading of new CENP-A during G1 in human cells (Fig. 4).

## The role of CENP-A in mitosis and its relation with cancer therapy

Many labs working in a myriad of model systems have concluded that centromere identity propagation and maintenance through CENP-A is essential for cell division. CENP-A localization to centromeres creates a platform that is essential for kinetochore assembly; a loss in centromere identity results in chromosome segregation defects caused by a misattachment of chromosomes to the mitotic spindle. To date, centromere proteins such as CENP-A and KNL-2 have no known role outside of mitosis, thus making them appealing targets for chemotherapeutic development. Inhibiting centromere function leads to chromosome segregation defects and ultimately to cell death, thus possibly a potent mechanism for slowing down cancer progression. Importantly, inhibiting mitosis-specific mechanisms will diminish the possibility of undesirable side effects as seen in taxol treatment. This broadly used chemotherapeutic treatment stabilizes microtubules leading to mitotic defects and cell death. However, microtubules also have essential roles as cytoskeleton components for most cell types, including neurons. Therefore, taxol also affects microtubules in the nervous system resulting in severe undesired side effects usually accompanied by neurodegenerative pathology. Thus, investigations with the aim of elucidating how the cell manages to preserve its centromeres well defined during cell division will likely provide us with new drug targets with higher specificity for mitotic cells.

Centromere fields of research are expanding, trying to better understand the complex cellular process of centromere identity and maintenance. In the next years, our efforts will bring more highlights to the spatial and temporal regulation of CENP-A loading to centromere, thus better define the first marks of centromere identity.
